# Invasive species trait-based risk assessment for non-native freshwater fishes in a tropical city basin in Southeast Asia

**DOI:** 10.1371/journal.pone.0248480

**Published:** 2021-03-16

**Authors:** Joleen Chan, Yiwen Zeng, Darren C. J. Yeo

**Affiliations:** 1 Department of Biology Sciences, National University of Singapore, Singapore, Republic of Singapore; 2 Lee Kong Chian Natural History Museum, National University of Singapore, Singapore, Republic of Singapore; Instituto Federal de Educacao Ciencia e Tecnologia Goiano - Campus Urutai, BRAZIL

## Abstract

Biological invasions have created detrimental impacts in freshwater ecosystems. As non-native freshwater species include economically beneficial, but also harmful, species, trait-based risk assessments can be used to identify and prevent the import of potentially invasive species. Freshwater fishes are one of the most evaluated freshwater taxa to date. However, such assessments have mostly been done in sub-temperate to temperate regions, with a general lack of such research in the tropics. In view of this knowledge gap, this study aims to determine if a different set of traits are associated with successful establishment of non-native fishes within the tropics. In tropical Southeast Asia, Singapore represents a suitable model site to perform an invasive species trait-based risk assessment for the tropical region given its susceptibility to the introduction and establishment of non-native freshwater fishes and lack of stringent fish import regulation. A quantitative trait-based risk assessment was performed using random forest to determine the relative importance of species attributes associated with the successful establishment of introduced freshwater fishes in Singapore. Species having a match in climate, prior invasion success, lower absolute fecundity, higher trophic level, and involvement in the aquarium trade were found to have higher establishment likelihood (as opposed to native distributional range and maximum size being among the commonly identified predictors in subtropical/temperate trait-based risk assessments). To minimize invasive risk, incoming freshwater fishes could be screened in future for such traits, allowing lists of prohibited or regulated species to be updated. The findings could also potentially benefit the development of invasive species action plans and inform management decisions in the Southeast Asian region. Considering a geographical bias in terms of having relatively less documentation of biological invasions in the tropics, particularly Asia, this study highlights the need to perform more of such risk assessments in other parts of the tropics.

## Introduction

The human-mediated translocation of species outside of their native range has led to the proliferation of invasive species globally, causing widespread abiotic and biotic changes [1] through the alteration of ecosystems [2] and loss of native species [3]. Human health and economy have also been affected due to the transmission of diseases, reduction of crop yield, and costs to control invasive species [1]. These effects are especially pronounced within freshwater ecosystems, owing to the intensive and extensive use of non-native species for recreation and food provisioning, and to accidental transport [4, 5]. Such pathways have led to the establishment and spread of harmful aquatic invasive species through release of unwanted aquarium pets [6, 7] deliberate stocking of lakes for sport fishing or aquaculture [8, 9], and unintentional introductions from ship ballast water [10, 11]. Considering the greater ease of dispersal (resulting from the connectivity and flow of water within freshwater systems) and higher endemism, freshwater ecosystems are particularly vulnerable to biological invasions compared to terrestrial ecosystems [4, 12].

Before causing any substantial harmful effects in a new environment, introduced species must pass through several invasion stages and overcome anthropogenic, biological, and environmental barriers [13, 14]. This process first involves a species being transported and introduced into an area outside of its native range, then surviving and reproducing to form an established or self-sustaining population before spreading to other habitats [14]. These species are then only considered invasive when they cause net harm to the environment, native biodiversity, economy, or human health [1, 15]. However, not every species is able to overcome the barriers associated with each stage of the invasion process, and even if they do, such species can turn out to be benign (e.g., [16, 17]).

Given the potential economic benefits (e.g., recreational sport fishing and aquarium pets; [7, 18, 19]), but at the same time devastating and costly impacts associated with invasive freshwater species [15], a cost-effective approach is needed to prevent the import of potentially invasive species while allowing benign species to enter [20]. Consequently, invasive species risk assessment methods have been developed to assess the likelihood of particular species to become invasive if ever introduced [21]. Such assessments range from the of semi-quantitative screening kits (e.g., Fish Invasiveness Screening Kit; [22]) to quantitative statistical analyses such as modelling of propagule pressure and ecological niche (e.g., [23–25]) and trait-based assessments (e.g., [26, 27]). While many methods exist to assess the risk of a non-native species (to become invasive), trait-based risk assessment remains one of the most popular techniques available (e.g., [27–30]). Through the quantification of biological and ecological species traits that are associated with a species’ ability to overcome barriers associated with each invasion stage [20], trait-based risk assessment provides a straightforward and accurate means of identifying and predicting high- and low-risk species [21, 31]. As such, trait-based risk assessment has been performed for numerous freshwater taxa (e.g., fish, crayfish, and molluscs [26, 27, 32]), at both local (e.g., [26, 33]) and global scales (e.g., [29, 34, 35]).

Focusing on multiple aspects of a species’ biology, including life history (e.g., fecundity [32] and egg size [36]) and physiological tolerance [30], as well as indicators of a species’ ecology, such as distributional range or distance to source [37], trait-based risk assessments identify a species’ innate likelihood of succeeding at an invasion stage in an introduced environment [21]. These risk assessments also allow users to account for anthropogenic factors through the use of variables such as propagule pressure [37] or human-uses [27]. Following a compilation of studies that conducted quantitative analyses of trait-based risk assessment on various freshwater taxa (see S1 Table; an earlier study by García-Berthou offers a compilation on freshwater fishes [38]), a few trends were brought to light. Of the freshwater taxa examined, fishes appeared to be the most studied taxon, likely due to their ecological [39] and economic [18, 19] importance. Among the species attributes or traits analysed, maximum size, fecundity, and native distributional range appear to be among the most commonly identified predictors of both establishment likelihood and invasiveness of freshwater species ([27, 30, 32, 33, 35–37, 40–47]; S1 Table). However, although trait-based risk assessments have been performed at varying geographic scales, local assessments have mostly focused on the sub-temperate to temperate regions of the world ([48]; S1 Table). With a relative lack of research efforts in the tropics [49, 50], and the potential variation in identified traits at the local level ([51]; see also S1 Table), it is unclear if risk assessments performed within tropical regions would identify similar patterns or suites of traits as those conducted in other climatic regions.

Therefore, in light of this paucity of information, and a noted difference in invasibility of tropical ecosystems [52, 53], this study aims to determine if non-native species within tropical climates are associated with a different set of traits in overcoming the barriers in the invasion process. To do so, we performed a trait-based risk assessment with a focus on the establishment stage of introduced freshwater fishes, one of the most well studied freshwater groups (S1 Table), within Singapore, a country in tropical Southeast Asia.

## Materials and methods

### Study site

**Fig 1:**
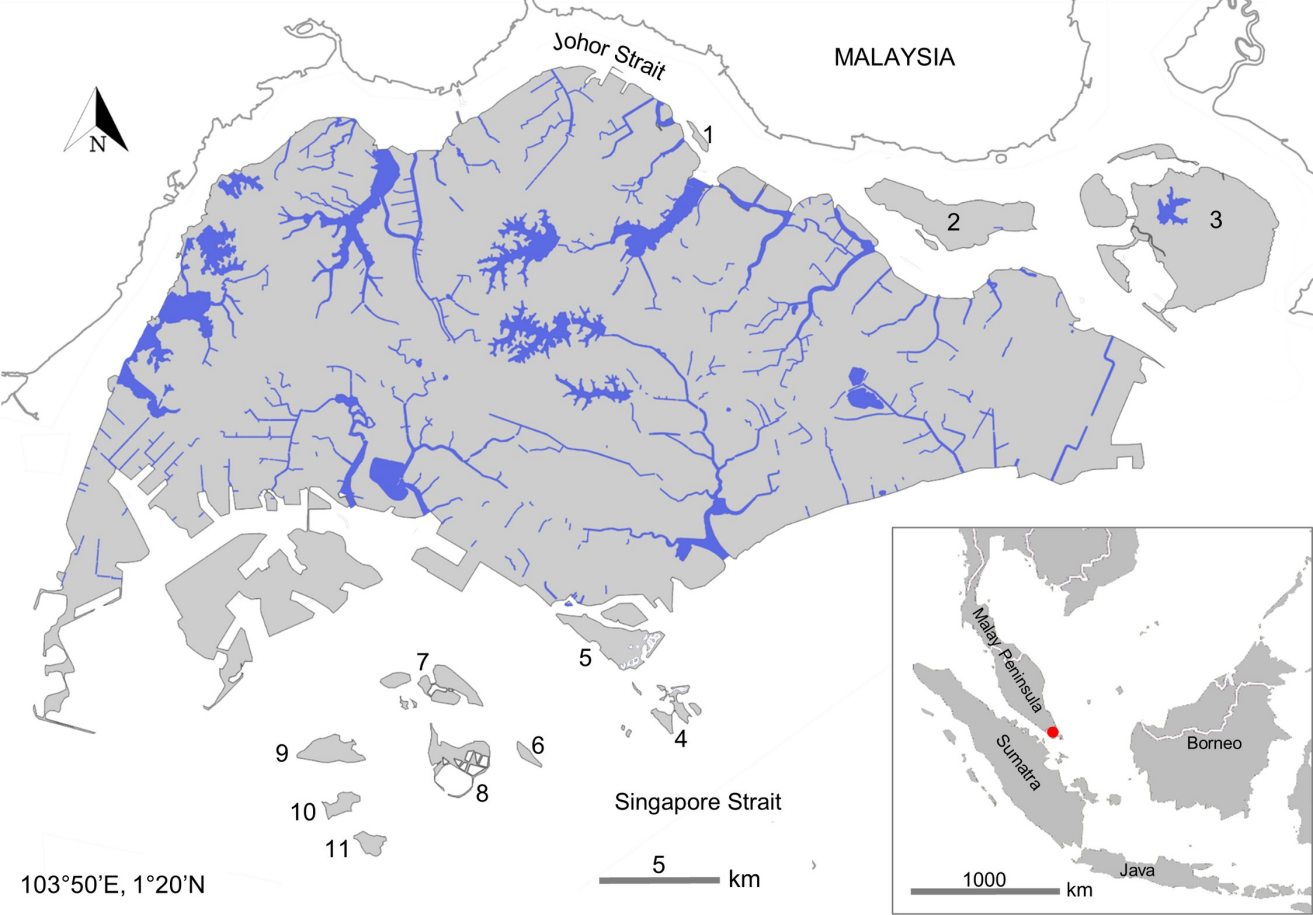
Map showing Singapore’s freshwater environment, which comprises a highly inter-connected network of man-made reservoirs, rivers, streams, canal and drains. 1. Pulau Seletar, 2. Pulau Ubin, 3. Pulau Tekong, 4. St. John’s, Lazarus and Kusu islands, 5. Sentosa, 6. Pulau Sebarok, 7. Pulau Bukom, 8. Semakau Landfill, 9. Pulau Sudong, 10. Pulau Pawai, and 11. Pulau Senang.

The region of risk assessment, Singapore (103°50’E, 1°20’N), covers a small geographical area of about 720 km^2^, and experiences an equatorial tropical climate with high humidity and slight seasonality in rainfall throughout the year, largely driven by the monsoon [54, 55]. The freshwater environment of Singapore (Fig 1) comprises both natural and artificial ecosystems, with forest streams and freshwater swamps serving as strongholds for native fishes, while man-made reservoirs and canals are dominated by non-native fishes [56, 57]. Most of the original river systems have been modified through channelization and damming for flood control and construction of reservoirs [56]. These freshwater bodies are highly connected for water management purposes [58], hence all freshwater habitats were considered in this assessment, ranging from streams and rivers to urban reservoirs, ponds, and drainage networks.

As one of the world’s busiest ports and largest exporter of ornamental fishes [59, 60] with a lack of strict regulation of fish imports [61, 62], Singapore is particularly susceptible to the introduction of non-native freshwater fishes. Such risks have been increasingly apparent as indicated by growing records of introduced freshwater fish species in Singapore (e.g., [57, 62–67]). Many of these fishes are unwanted ornamental fish released into publicly accessible artificial water bodies (i.e., aquarium dumping) that constitute a large proportion of Singapore’s waterways [57, 65]. As a result, the number of introduced freshwater fishes rose from just eight in the 1960s [63] to as many as 123, over 50 years—more than a third of which were found to have established [57]. As such, taking all freshwater environments in Singapore into consideration in this study (due to the small area and their high connectivity), we populate a list of non-native freshwater fish species for analyses.

### Attribute database of non-native freshwater fish species

A list of 98 non-native freshwater fish species in Singapore was compiled by referring to relevant literature (e.g., [57, 62, 65, 68–70]), which spanned from 1849 to the conclusion of this study in 2016. Species were included on the basis that they were recorded as introduced species in any freshwater bodies of Singapore.

To determine the invasion status of the fish species (established or failed to establish) in Singapore, we referred to Tan et al. [57] which provides the most current record. Species that have not established after 15 years since their first record of introduction were assumed to have failed to establish (following [71]).

In order to establish, a species would have to overcome the challenges of surviving and reproducing successfully to produce viable offspring and form self-sustaining populations in a new environment [14]. Thus, 21 species attributes (explanatory variables) believed to be correlated with successful survival and reproduction (and therefore establishment) in a foreign environment were analysed (see S1 File for details). They include 14 ecological, biological, and behavioural attributes that potentially determine establishment success. Ecological traits include (1) habitat type (lentic, lotic slow, lotic fast, both lentic and lotic); (2) habitat generalist (yes, no); (3) vertical position (benthopelagic, pelagic, demersal); (4) habitat salinity (freshwater, both fresh and brackish water); (5) climate match (yes, no; determined using the Köppen-Geiger climate map [72]); (6) climate types, (number of climate types a species can survive in, determined using the Köppen-Geiger climate map [72]); and (7) trophic level (values obtained from Fishbase [73]; ranges from 2.0 to 4.7). Biological traits include: (8) maximum standard length; (9) absolute fecundity (greatest number of mature oocytes present in a fish prior to a spawning event); (10) mode of reproduction (oviparous, ovoviviparous); (11) parental care (non-guarders, guarders, bearers [74]); (12) diet (herbivorous, omnivorous, carnivorous); (13) air-breathing (yes, no); and (14) schooling behaviour of adults (yes, no). Four human-use attributes considering the motivations for the import of non-native fishes were also included—use of fishes in aquarium, aquaculture, angling, and biological control (each has a binary response of yes or no). In addition, year of introduction (year a species was first recorded in the wild in Singapore), and invasion history (yes, no) were also taken into account. Family was also added in the analysis in order to account for the non-independence between species due to phylogenetic relatedness as well as to determine the importance of family as a predictor. Several more attributes were considered initially (e.g., gonadosomatic index, egg diameter, reproductive age, and lifespan), but were eventually omitted owing to the lack of information.

Information on these 21 attributes was gathered from published literature and online databases such as Fishbase [73] and Invasive Species Specialist Group‘s Global Invasive Species Database, ISSG-GISD (http://www.issg.org/database). Theses and field guides were also referred to. Reliable reference websites, such as Seriously Fish (www.seriouslyfish.com; as used in other studies, e.g., [75]), were also referred to as supplementary information sources for some species traits. Aquaculture or fisheries studies that involved the use of hormones to induce spawning or rapid growth were intentionally disregarded, as the use of hormones may lead to inaccurately high fecundity. In cases where multiple data points were available for a species’ specific trait, we reported the greatest value recorded in scientific literature as an indication of the species’ maximum potential (following [27]).

### Data analysis

Three life history attributes—maximum standard length (mm), absolute fecundity, and parental care—were assessed qualitatively to determine if the successfully established non-native species possess distinct life history strategies as proposed in the Winemiller and Rose triangular life history model, which recognised three strategies: (i) opportunistic (small body size, early maturation, high reproductive effort, low batch fecundity, and low degree of parental care); (ii) periodic (large body size, delayed maturation, moderate reproductive effort, high batch fecundity, and low degree of parental care); and (iii) equilibrium (variable body size, moderate maturation period, low reproductive effort, low batch fecundity, and high degree of parental care) [76].

To identify the attribute and determine their relative importance in predicting establishment (response variable), an ensemble method, random forest (RF; [77]) was employed. Utilizing a large number of decision trees in the classification and prediction of a dataset, RF presents a more reliable and robust method than the use of a single tree [78]. Random forest was preferred to other statistical techniques such as Generalised Linear Mixed Models as it is more robust for small datasets that contain several explanatory variables [21, 79] such as in this study (60 species, 21 species attributes).

Each tree in the RF models was constructed using a non-parametric recursive binary partitioning technique known as Conditional Inference Tree (CIT; [80]). Conditional Inference Tree was used instead of the more commonly used Classification and Regression Tree as it avoids both issues of overfitting and selection bias towards variables with many possible splits or missing data [80]. In this conditional inference framework, overfitting and the subsequent need for pruning are avoided as a stopping criterion is applied to halt further partitioning or growing of the tree. This is accomplished using standardized linear statistics that assess the null hypothesis of independence between the response and explanatory variable. If the p-value is greater than 0.05, the null hypothesis is not rejected and the data partitioning discontinues. In this way, species attributes that are strongly associated with successful establishment will be identified and used in the construction of the decision trees. In this study, Monte Carlo P <0.05 was employed and a minimum splitting criterion of two was selected. As the procedures of variable selection and splitting process were done separately using suitable statistical tests, bias in variable selection was avoided [80]. In CIT, a subset of explanatory variables were randomly selected as candidates at each node (mtry = 7 in this study), thereby allowing all explanatory variables to have a chance to be represented in the ensemble [81]. This enabled any interactions between variables, which would otherwise have been overlooked, to be detected [81].

With RF constructed, the relative importance of each explanatory variable was determined by computing the variable importance using the conditional permutation method [81, 82] so as to avoid any bias towards correlated explanatory variables. Each CIT was built using a bootstrapped sample of the original dataset, and cases not included in the bootstrapped sample formed the out-of-bag sample that was used to assess the misclassification rate of the tree. Following a random permutation of an explanatory variable in the out-of-bag sample, the association of the variable with the response variable was disrupted, enabling a misclassification rate to be obtained when this permuted sample was run down the classifier tree [78]. The difference in misclassification rates before and after permuting the variable was then averaged over all trees (n = 1000 in this study) to obtain the variable importance value. The greater the increase in misclassification rate, the more important the variable is in explaining the dataset [81].

As the attributes year of introduction and invasion history could potentially mask/confound the establishment likelihood of newly introduced species, an additional RF analysis was done without these attributes to assess the importance of other attributes subsequent to their removal. As invasive species risk assessments serve to evaluate the invasive risk of a species prior to its introduction, the attribute year of introduction cannot serve as a predictor, as such data are only available after a species has been introduced [21]. The attribute invasion history was removed due to the uneven research efforts across geographical regions with most biological invasions being documented in temperate regions [83]. Due to such geographical bias, not all species’ invasion histories have been documented. Moreover, some species were only reported as introduced for the first time and consequently have no invasion history [84]. Thus, the usefulness of invasion history as a predictor is reduced although it may potentially be a strong predictor of establishment.

Since the end of 2016, when the bulk of this study was completed, there were five new records of species establishment (see S1 Data), two of which (*Apistogramma borellii* and *Betta splendens*) were previously recorded as non-established. To assess model reliability, the full (21 attributes) and reduced (without attributes year of introduction and invasion history) RF models were reconstructed with the exclusion of these two species to predict the establishment likelihood of recently established species. The variable importance of the species attributes was also re-evaluated to assess if the omission of the two species would lead to a different set of attributes identified to be important in establishment success.

To assess the performance of the RF models, Receiver operating characteristic (ROC) curves were constructed. The ROC curve assesses both the true positive rate and the false positive rate of a model using varying thresholds, and the area under the ROC curves (AUC) was computed to obtain the performance of the models [85]. An AUC of 0.5 indicates that the model performs no better than chance as the true and false positive rates are equal whereas an AUC of 1.0 indicates a perfect discrimination of established and failed to establish species [86]. To reduce circularity, an additional AUC was computed using a training and test sample. Derived through stratified random sampling of the full dataset inclusive of all 21 species attributes [87], 70% of the dataset was used as the training sample, and the remaining dataset was used as the test sample to assess the AUC of the model.

All statistical analyses were done on R version 3.1.2 [88]. Random Forest analyses were performed using the package ‘party’ version 1.0–20 [80, 82, 89, 90]. The package ‘caret’ version 6.0–41 [87] was used to generate the training and test samples, and the package ‘pROC’ version 1.7.3 [91] was used to assess the AUC of RF models.

## Results

At the time this study was completed (end of 2016), a total of 98 freshwater fish species from 25 different families (see S2 File for summary) were recorded to have been introduced into Singapore’s freshwater habitat. Almost half of the total species reported were from two families, Cyprinidae (25.5%) and Cichlidae (23.5%), which similarly dominated the established species, constituting more than 57.4% of the species that have formed breeding populations in Singapore’s freshwaters. Of the 98 introduced species, 23 species were only introduced less than 15 years ago and had not been documented to have established. Thus, these species were not considered in this study. Of the remainder, a total of 60 species had complete data for all species attributes assessed (S1 Data), of which 36 are established and 24 have failed to establish (S2 File).

Based on the qualitative assessment of three life history attributes (maximum standard length, absolute fecundity, and parental care; see Fig A–C in S2 File), the established and non-established fishes do not seem to differ in body length. However, successfully established species generally have relatively lower fecundity and high parental care investment, indicative of an equilibrium strategy. Conversely, species that failed to establish appear to have a periodic strategy given the relatively higher fecundity and lower degree of parental care investment.

**Fig 2:**
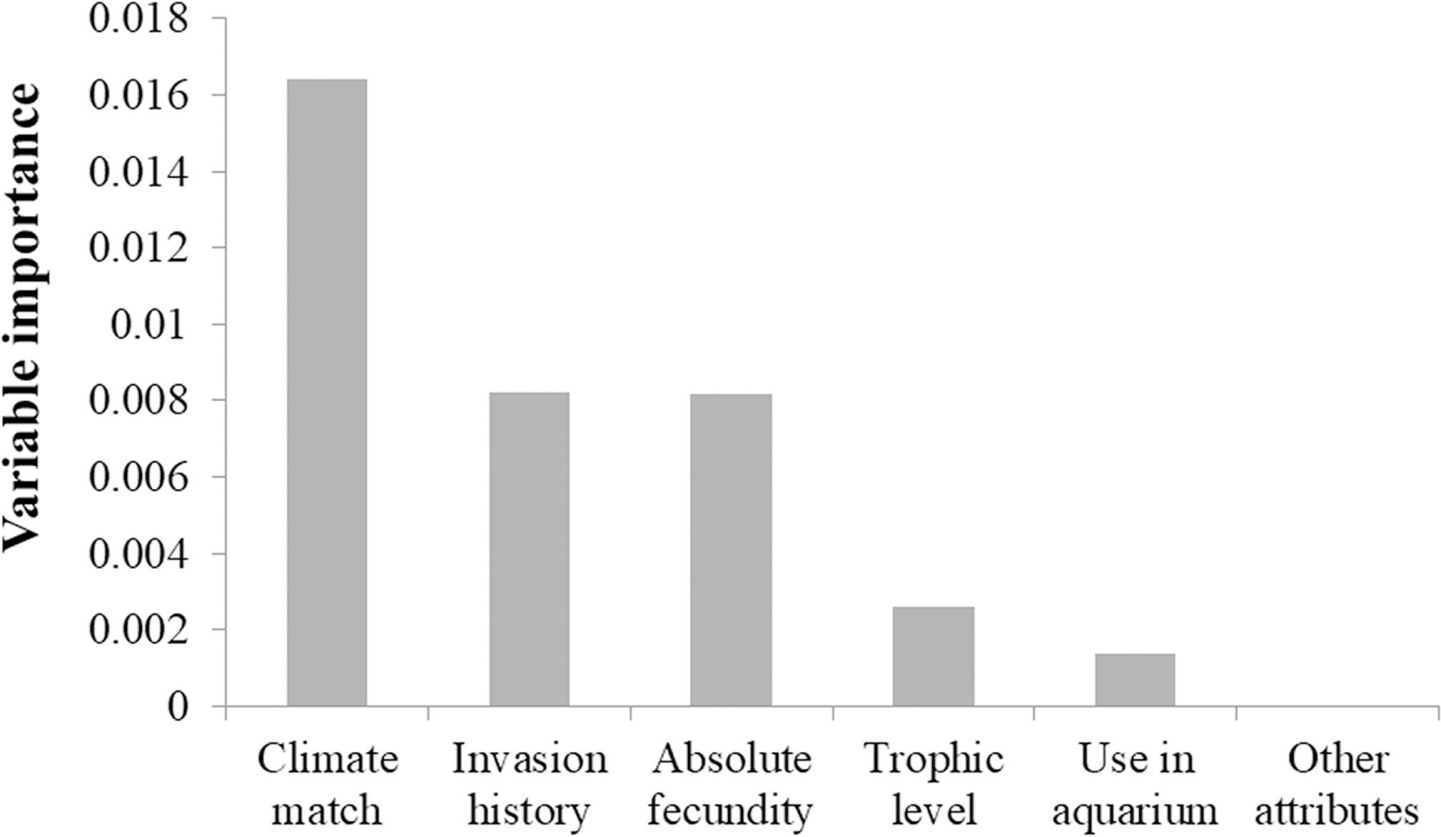
Relative variable importance of attributes (other attributes had relatively low variable importance and fluctuated about zero).

**Fig 3:**
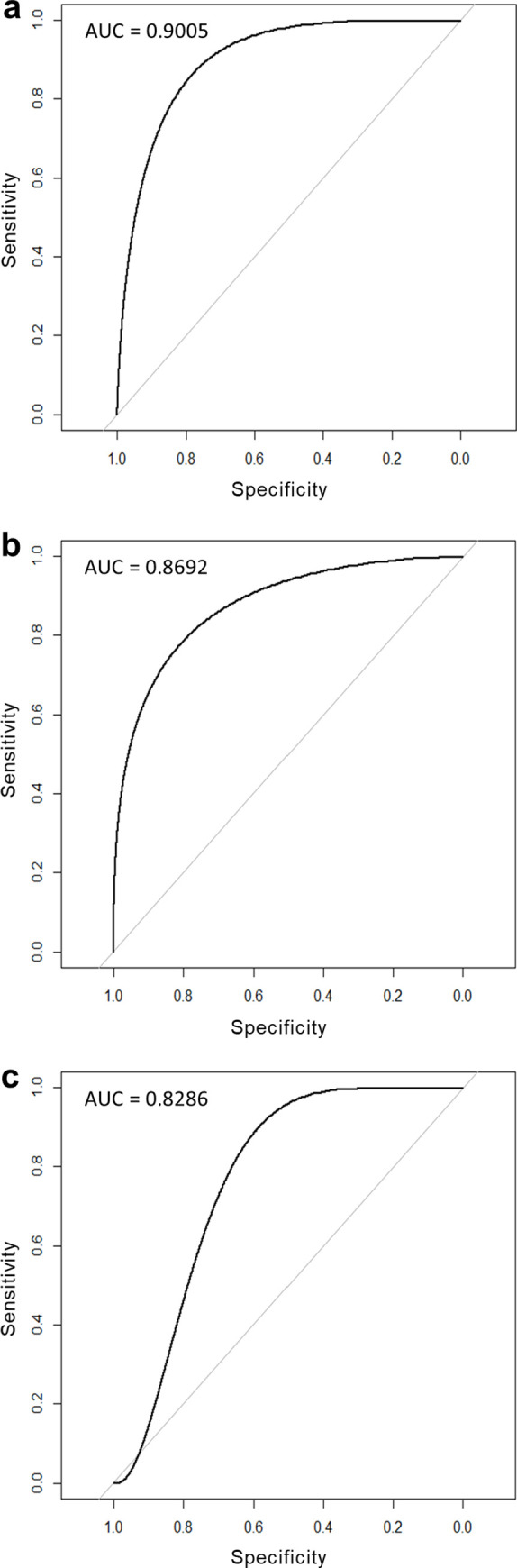
Receiver operating characteristic curves for (a) the full random forest model containing all 21 species attributes, (b) reduced random forest model without attributes year of introduction and invasion history, and (c) the training and test samples used to account for circularity.

The variable importance values obtained from the RF analysis indicated a relatively high importance of five attributes—climate match, invasion history, absolute fecundity, trophic level, and use in aquarium trade—in predicting for establishment (Fig 2). The RF model containing all 21 attributes performed well with an AUC value of 0.9005 (see Fig 3A for ROC curve). Accounting for circularity using the training and test samples yielded a similarly high AUC value of 0.8286 (see Fig 3C for ROC curve).

An examination of the five species attributes identified via variable importance revealed the characteristics that are potentially associated with successful establishment—having a native range with climate that matches that of Singapore (equatorial tropical climate), possessing prior invasion success, lower absolute fecundity, higher trophic level, and involvement in the aquarium trade likely contribute to successful establishment in Singapore (Table 1). On average, established species have a mean absolute fecundity value ten times lower than that of species that have failed to establish. In addition, species higher in the food web, being mainly carnivorous secondary consumers (trophic level >2.8) were more likely to establish than omnivorous species (trophic level 2.2–2.79; see [73] for trophic level classification).

**Table 1:** Attributes identified to be important predictors of establishment in random forest model. Standard errors are given for continuous data in parentheses.

Attributes	Established (n = 36)	Failed to establish (n = 24)
% Yes for climate match	72.2%	37.5%
% Yes for invasion history	75.0%	50.0%
Absolute fecundity	27,653 (12,362)	339,633 (129,476)
Trophic level	3.11 (0.105)	2.73 (0.111)
% Yes for aquarium	88.8%	70.8%

The reduced RF model that excluded the attributes year of introduction and invasion history showed that climate match, absolute fecundity, and trophic level were still the most important predictors of establishment (AUC value = 0.8692; see Fig 3B for ROC curve).

**Fig 4:**
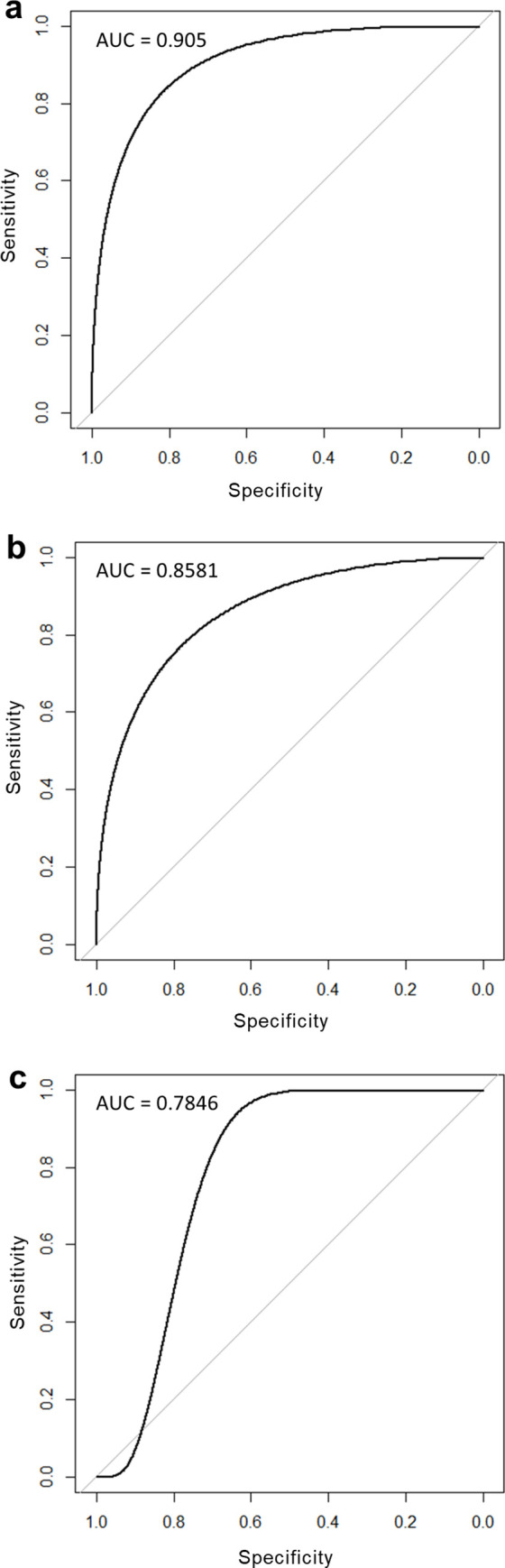
Receiver operating characteristic curves for models constructed to assess model accuracy in predicting for establishment likelihood. (a) The full random forest model containing all 21 species attributes. (b) Reduced random forest model without attributes year of introduction and invasion history. (c) The training and test samples used to account for circularity.

The full and reduced models constructed to test model reliability based on the recently established species (reported after 2016) had high accuracy, with all five species correctly predicted to establish (see Table D in S2 File). With the omission of two of these species previously recorded as non-established, the variables identified to be important in establishment success largely matched those of the initial models—climate match, absolute fecundity, and trophic level were found to be the three most important variables (see Table C in S2 File). Both full and reduced models performed well with similarly high AUC of 0.905 and 0.8581 respectively (see Fig 4A and 4B for ROC curves). The training and test samples yielded a relatively high AUC of 0.7846 (see Fig 4C for ROC curve).

## Discussion

The RF analysis of 21 attributes to determine variable importance identified five attributes that are associated with successful establishment. Climate match was the most important trait, followed by invasion history, absolute fecundity, trophic level, and use in the aquarium trade. The identification of climate match, absolute fecundity, and trophic level as the three most important variables consistently in all full as well as reduced (excluding the variables invasion history and year of introduction) RF models suggests that these attributes are strong predictors of establishment success. These factors probably enhance species establishment likelihood by contributing to their ecological and physiological adaptations [24, 32, 92, 93] and life history adaptations [46]. Furthermore, both full and reduced RF models that were used to assess model reliability correctly predicted the establishment outcome of the recently established species although fecundity data was absent for two species. Within the two dominant families (Cyprinidae and Cichlidae), established species on average indeed appear to have qualities of the five attributes important for successful establishment (see Table E in S2 File). Although the cyprinids tended to have higher fecundity and all cichlids provide parental care (see S1 Data), cyprinids and cichlids that established successfully generally possess lower fecundity than those that failed to establish.

The importance of climate match concurs with studies in temperate regions in which species introduced into an area of the same climate as their native range are likely to be more physiologically suited to that environment [28, 34, 94]. Whereas, the negative relationship between fecundity and establishment likelihood differed from most temperate studies, which generally identified a positive association [30, 40, 42] (although one study by Grabowska and Przybylsk (2015) conducted in the Central European bioregion did record a negative relationship [47]). This pattern could be a result of the negative correlation between egg size and fecundity [95]. Furthermore, the comparison of life history traits suggested that successfully established species in this study tend to have an equilibrium strategy, which is principally the K-selected strategy of high investment in individual offspring at the expense of fecundity [76]. It is thus possible that K-selected traits are generally more beneficial for establishment within tropical regions although R-strategists such as several characid and cyprinid species have also succeeded in establishing (e.g., [57, 96]). Considering the fact that K-strategists (such as *Poecilia reticulata* and *Oreochromis mossambicus*) produce fewer but larger and more developed offspring that are more likely to avoid predation and survive [74, 95], a lower absolute fecundity could therefore reflect a higher chance of survival and reproductive success within tropical environments. This is especially considering trophic food webs in tropical freshwater ecosystems are generally more complex than those in temperate regions due to the diversity of food resources and highly diverse feeding niches of tropical freshwater fishes [93, 97]. Often dominated by omnivorous fishes and comprising smaller-sized carnivorous fishes [98, 99], tropical freshwater environments may present a greater likelihood of predation of smaller and less developed fry or juveniles [100]. Thus, by channeling resources into producing fewer but larger eggs that hatch into more developed offspring, fishes with lower fecundity and high parental care investment may have greater establishment likelihood in tropical freshwater ecosystems. Such a negative association between fecundity and establishment success has also been observed when identifying traits associated with establishment likelihood of non-native fishes on a global scale [46].

A difference in establishment likelihood in tropical and temperate freshwater ecosystems is illustrated by the importance of trophic level as a predictive trait found in this study. Although trophic status or level is commonly identified as an important predictor of multiple invasion stages in temperate regions, these studies only found it to be important in post-establishment stages (S1 Table). Furthermore, only one study (done on non-native fishes in the Great Lakes using trait-based classification trees) showed a correlation between higher trophic levels and invasion likelihood [28]. These differences could arise from the fact that a high proportion of tropical freshwater fishes tend to be herbivorous, detritivorous, or omnivorous, and proportionally fewer are large piscivorous fishes, compared to temperate freshwater fish assemblages [93, 98, 99]. It therefore may be possible that more predatory fishes (i.e. higher trophic level) could take advantage of the relatively less diverse and occupied piscivory niche in tropical food webs, thus favouring their establishment in tropical freshwater habitats. This is likely to be exacerbated by habitat disturbance (such as river impoundments) which creates vacant niches through loss of native species and provision of novel environmental conditions [101].

Besides the difference in how some of these identified traits are associated (positively or negatively) with establishment likelihood, the relative importance of these variables also appears to differ in tropical ecosystems. While other studies usually identify invasion history as the key predictor associated with success at most stages of the invasion process for non-native freshwater fishes [15, 26, 35, 38, 41], this study identified climate match to be more important. This is likely because most invasions have been documented in temperate regions (particularly North America, Europe, and Australia), and less so in tropical Asia and Africa [83]. Thus, many biological invasions in the tropics may have been overlooked and gone undocumented as suggested by recent detections of potentially invasive species in tropical lentic ecosystems (e.g., [102, 103]) using eDNA techniques, which have so far been more widely employed in temperate freshwater habitats [104]. Considering the importance of climate match, the effects of such geographical bias would be especially pronounced for tropical species.

Geographical bias is also manifest in the association of successful establishment with the use of species in aquarium trade. Owing to the growing popularity of tropical aquarium fishes [105, 106], and the common practice of discarding unwanted pets [6, 7, 106] (or religious release of ornamental fishes in countries such as Singapore [62] and Taiwan [30]), the aquarium trade has gained importance as an invasion pathway in many countries (e.g., [105, 107, 108]). In addition, aquarium trade species are often associated with a preselection of ecological traits (e.g., climate match, reproductive potential, and environmental tolerance) that increases the chance of survival within other tropical habitats [27]. This, coupled with the popularity of ornamental fishes and their eventual introduction (increased propagule pressure), results in a greater probability of establishment [109].

Given that majority of the introduced species are aquarium fishes (>75% of the species compiled in this study), quantitative or semi-quantitative risk assessments/tools can potentially be adopted by authorities as a pre-import initial risk screening routine as recommended by Yeo and Chia [62]. Such measures could help identify potentially invasive fish species prior to their import. Such high-risk species could also be added to lists of prohibited or regulated fishes that prevent or control the import, sale, and release of potentially invasive ornamental species. Currently, only piranhas (Actinopterygii: Serrasalmidae) are specifically prohibited in Singapore under the Animals and Birds (Piranha) Rules 2019 [110]. Importantly, regulation should also be coupled with education to increase public awareness of the potential adverse effects of releasing non-native species into the waterways [62].

The potential geographical biases associated with trait-based risk assessments within the tropics, and the differences from several temperate studies in association (positive or negative) for some key traits revealed in this study, indicate that there is a clear need to perform more of such risk assessments for the tropics (e.g., [111, 112]). This is especially so considering the higher level of native species richness and endemism within tropical regions [113], which could indicate a greater or wider risk associated with the ecological impacts of invasive species. Given the major role that trade in freshwater fish plays in tropical Southeast Asian economies (e.g., aquaculture, aquarium trade [114] and the potential devastating economic impact invasive species could cause in the region [115], trait-based risk assessments can provide a means of evaluating and mitigating the risks of non-native fish species. Additionally, while predictive models of invasive species are often site-dependent [51], given the relative faunistic as well as climatic similarity between Singapore and other tropical environments within Southeast Asia (such as Peninsular Malaysia) [72, 116], findings from this study could be applicable to the region. Moreover, highly urbanized Singapore, with its significant freshwater habitat alteration and losses [117, 118] and high occurrences of non-native species [57, 119, 120] serves as comparable future environmental scenario that other Southeast Asian countries are rapidly developing towards. Therefore, findings of this trait-based risk assessment may be beneficial in the development of invasive species action plans in the wider region and informing policy/management actions by, for instance, streamlining existing lists of prohibited fishes (i.e. blacklists) (e.g., [121, 122]) or informing the formulation of such lists.

Undoubtedly, the constructed trait-based models in our study are limited by the availability of updated data such as documentation of newly established species and information on various species attributes. As more studies and data become available, model performance and predictions of establishment likelihood of non-native species would certainly improve. Furthermore, some species previously recorded as non-established might succeed with time (e.g., establishment of *A*. *borellii* and *B*. *splendens* after 2016), and models could be updated with such new documentations to keep them relevant. Nonetheless, the predictive accuracy of the current models is high, with the ability to predict correctly the five species recently recorded (between 2017 and 2020) to have established. This highlights the usefulness of trait-based risk assessments as a preemptive screening measure to assess a species’ potential risk of invasiveness.

## Conclusion

By performing a trait-based risk assessment of introduced freshwater fishes using Singapore as a model site for tropical Southeast Asia, we identified a similar but essentially unique set of traits important as predictors of establishment success compared to temperate regions—climate match being the most significant, followed by invasion history, absolute fecundity, trophic level, and use in aquarium trade. Although most of these traits were also found to be important predictors for establishment in temperate regions, the general dissimilarities in association of traits identified (i.e. lower fecundity and higher trophic level), and the relative importance of these traits, are a reflection of the intrinsic differences between tropical and temperate freshwater ecosystems as well as the geographical bias in documentation of biological invasions. In view of the difference in predictors identified and high predictive accuracy achievable, it is thus vital and beneficial to perform risk assessments for invasive species in the tropics, which would further our understanding of biological invasions in this climatic zone.

## Supporting information

S1 Table Species attributes found to be important determinants of establishment likelihood. (XLSX)

S1 File Detailed information of the attributes analysed in the trait-based risk assessment for non-native freshwater fishes in Singapore. (DOCX)

S2 File List of families of non-native freshwater fishes in Singapore, summary of data gathered for the 20 species attributes used in the trait-based risk assessment, variable importance values and predictions from Random Forest analyses. (DOCX)

S1 Data Database on non-native freshwater fish species and attributes analysed in the trait-based risk assessment. (XLSX)
