# Supplementary material for: Invasive species trait-based risk assessment for non-native freshwater fishes in a tropical city basin in Southeast Asia
Source: PLoS One. 2021 Mar 16;16(3):e0248480. doi: 10.1371/journal.pone.0248480 (PMC7963036; doi:10.1371/journal.pone.0248480)
Supplement: S1 File — (DOCX) [file pone.0248480.s002.docx]

**S1 File. Detailed information of the attributes analysed in the trait-based risk assessment for non-native freshwater fishes in Singapore.**

| Attribute | Variable type | Definition | Rationale | Reference |
| --- | --- | --- | --- | --- |
| Ecological |  |  |  |  |
| Habitat type | Categorical:  Lentic  Lotic slow  Lotic fast  Both | Flow speed of species’ native habitat. Considers suitability of the hydrological regime of Singapore’s freshwater environment for survival and establishment of a species. As flow speed of lotic systems varies between slow to fast, lotic species were classified as Lotic slow or Lotic fast. If species’ native habitat includes both lentic and lotic systems, then it was classified as Both. | Singapore’s freshwater environment consists mostly of artificial lentic and lotic water systems such as reservoirs and canals, thus species able to thrive in wide range of flow speed (i.e., Both) are more likely to survive and establish. Species able to survive in lentic or slow-flowing waters can also possibly establish in Singapore if they are introduced into habitats with slow-flow speeds (e.g., reservoirs). Already, most species which now thrive in Singapore’s reservoirs and ponds are non-native species adapted to sluggish conditions. Riverine species are unlikely to be able to establish as Singapore naturally lacks large river systems. | [1] |
| Habitat generalist | Binary | Yes: Species that is able to thrive in human-disturbed environment with low water quality and requires no specific environmental conditions to survive or reproduce. | Generalist species are able to tolerate disturbed environmental conditions, thus, more likely to thrive in artificial and human-disturbed habitats than species that require specific environmental conditions for survival. As Singapore’s highly modified and disturbed artificial waterways are often subjected to sudden increase in water volume in times of heavy rain or are polluted, generalist species would have higher potential to survive and establish. | [1, 2] |
| Vertical position | Categorical: BenthopelagicPelagic Demersal | Vertical position in water column in which a species usually occupies. Benthopelagic: Species that usually occupy near-bottom to mid-waters or near surface. Pelagic: Species that occupying midwater and near surface. Demersal: Benthic species | It is proposed that species that are able to inhabit wider vertical water column (i.e. Benthopelagic) will have a greater success in establishment as inhabiting larger habitat area gives them greater access to resources (such as food and refuge) which are important for survival and colonization. | [3] |
| Habitat salinity | Categorical: Freshwater Both | Accounts for the range of salinity in which a fish can survive in. Freshwater: Species which thrive primarily in freshwater. Both: Species that are able to survive in both freshwater and brackish. | Wide range of salinity tolerance has been attributed to successful establishment of introduced species, it is thus hypothesised that species classified as Both, are more likely to survive and establish in Singapore. Species with wider salinity tolerance would be more tolerant of frequent salinity fluctuations in Singapore’s reservoirs caused by monsoon seasons. Such species are also more likely to survive a stark change in salinity when released or escaped into the wild. | [4-6] |
| Climate match | Binary | Determined using the Köppen-Geiger climate map.  Yes: Species’ native range includes area with the same climate type as Singapore (i.e., tropical rainforest climate). | As species with good climate match is likely to be physiologically adapted to a foreign area, climate match has been widely recognized as an important predictor in many studies on non-native fish and other taxa. As climate influences the temperature of water bodies, which influences the successful survival and reproduction of fishes, species with a match in climate is more likely to establish. | [5, 7-10] |
| Climate type | Discrete | The number of climate types a species can survive in. Determined using the Köppen-Geiger climate map by manually counting the number of climate types that the native range of the species encompasses. When there is uncertainty if a climate type is included in a species’ native range, it was not counted. | Species that are able to tolerate a wide range of temperature and climatic variation were found to have greater likelihood of survival and establishment. Thus, it is expected that species with native range covering a greater number of climate types have higher chance of establishing in Singapore. | [4, 5, 7, 11] |
| Trophic level | Continuous | Data obtained from Fishbase which estimates trophic level using the Ecopath software^1^. Takes into account the diet composition of fish species as well as trophic level of food consumed. Derived by adding one (the default trophic level of primary producers and detritus) to the mean trophic level of food items weighted per proportion. Such estimates have been demonstrated to be highly correlated with estimates of stable isotope ratios. | Unlike the attribute Diet, considers the interaction of the species with other organisms in the food web. It has been observed that piscivorous or omnivorous fishes have a greater chance of establishing. Piscivores are possibly novel predators while omnivores, exhibiting a generalist diet, seldom experience limiting food resources. Thus, it is suggested that species with intermediate trophic level between 2.2 to 2.79 (omnivorous) or high trophic level >4 (top predators) are more likely to establish in Singapore. | [2, 5, 8, 12, 13] |
| Biological |  |  |  |  |
| Maximum standard length | Continuous | Largest documented standard length (measured in mm) attainable by a fish in the wild. Excludes cultivated fishes. | Most introduced freshwater fishes are discarded aquarium pets. As these fishes are often released when they outgrow their aquaria, it is likely that larger species have greater opportunity to be released into the wild than smaller-sized ones. With greater frequency of introduction or propagule release, establishment is more likely. More importantly, larger species are less vulnerable to predation and also likely to have higher fecundity and longevity. Thus, large-bodied species are likely to establish. | [10, 14-18] |
| Absolute fecundity | Discrete | Greatest number of mature oocytes present in a fish prior to a spawning event. For live-bearers, the maximum number of live young documented to have been produced is recorded. | Fecundity has been shown to be a good predictor of successful establishment in many studies. Conferring advantages of fast colonization as an r-selected trait, species with high fecundity are more likely to increase greatly in population size. This leads to a faster rate of propagule production which translates to a greater probability of establishment. Therefore, it is suggested that species with high fecundity are more likely to establish in Singapore. | [8, 9, 19-21] |
| Mode of reproduction | Categorical: Oviparous Ovoviviparous | Oviparous: Species that produces eggs that hatch outside the body. Ovoviviparous: Species that give birth to live young. | Ovoviviparous species are able to proliferate and increase in numbers rapidly; it is thus likely that such reproduction mode confer competitive advantage. Live bearers could multiply rapidly even when introduced in low numbers as a single female can produce multiple broods. Such embryos are protected from predators and fluctuations in environmental conditions. Born well-developed, their likelihood of survival and reproduction is higher. | [22-24] |
| Parental care | Categorical: Non-guarders Guarders Bearers | Following the classification of reproductive guilds outlined by Balon (1990). Non-guarders: Species that do not provide any form of parental. Guarders: Fend eggs from potential predators, build nesting sites, and/or tend clutch by fanning. Bearers: Either internal (live bearers) or external (e.g., mouth-brooding species) brooders. | Considered in many studies as predictor of successful establishment. Study by Marchetti et al. (2004), which had also adopted similar categories, found parental care to be important in determining success at the establishment stage. By caring for their eggs/embryos, the survival of offsprings is enhanced and chance of dispersing into environments that endanger them is lower. Thus, it is hypothesised that species with parental care (Guarders or Bearers) are more likely to survive and establish. | [4, 10, 19, 24, 25] |
| Diet | Categorical: Herbivorous Omnivorous Carnivorous | Herbivorous: Feeding primarily on plant, detritus and/or phytoplankton. Omnivorous: Feeding on other animals and plant.Carnivorous: Feeding on other animals. | Although categories used across studies vary, it has been shown that species with wider diet breadth or omnivorous are more likely to establish as generalist feeders are able to utilize a wider range of food types. Thus, it is hypothesised that omnivorous species are more likely to establish. | [4, 8] |
| Air-breathing | Binary | Yes: Ability to breathe atmospheric air. | Air-breathing confers advantages to species living in environments with low amount of dissolved oxygen. Thus, likely to assist survival in harsh environments of Singapore’s waterways. Due to high temperatures and relatively low wind speed in the tropics, Singapore’s reservoirs are susceptible to weak thermal stratification and low dissolved oxygen, which can result in mass fish death. Thus, it is suggested that species capable of air-breathing is more likely to establish in Singapore. | [1, 26-28] |
| Adult gregarious | Binary | Yes: Adults exhibit schooling behavior with conspecifics. | Behavioural attributes as potential predictors of successful establishment have often been overlooked in many invasive species behavioural studies. However, gregariousness in fishes has been considered to be predictor of establishment. Beneficial to survival and establishment as it contributes to greater ease of finding mates, and protection from predator by being in numbers. Species fitness is reduced when population size and density are small (Allee effect), thus aggregations facilitates successful establishment. Solitary species reduce their population density, thus more susceptible to Allee effects. It is proposed that gregarious species are more likely to survive and establish breeding populations. | [20, 29-33] |
| Human-use |  | Both past and current local uses were considered. A few species had unknown origins and human uses. | Considers the motivations for the import of non-native fishes in Singapore. Species affiliated to humans likely have higher success rate of establishment due to pre-selection of traits. Species deemed to be able to survive are more often introduced than those predicted to fail. | [34, 35] |
| Aquarium | Binary | Yes: Available in ornamental trade as feeder fish or pet fish. | Singapore is one of the largest exporters of ornamental fishes, and most introduced fishes originated from the aquarium trade. Such fishes are often released intentionally by fish hobbyists or through religious practices. It is thus hypothesised that aquarium fishes have higher likelihood of establishment due to greater propagule supply. | [14, 15, 36, 37] |
| Aquaculture | Binary | Yes: Cultivated for food or for aquarium trade. |  |  |
| Angling | Binary | Yes: Used for angling or deliberately released into the wild for sport fishing |  |  |
| Biological control | Binary | Yes: Used for controlling the abundance of other organisms. |  |  |
| Human-associated |  |  | Have been recognized as useful predictors of establishment. | E.g., [4, 18] |
| Year of introduction | Discrete | The year a species was first recorded in the wild in Singapore. | As lag time exists between stages of the invasion process, it is proposed that species introduced earlier is more likely to have established. A longer introduction history also meant that the species has more time for subsequent propagule releases to occur, thus higher chance of establishment. | [8, 38] |
| Invasion history | Binary | Yes: Species has a documented invasion success elsewhere. | Many studies found that species with prior invasion success were more likely to succeed in establishment stage. Invasive fish species tend to be widely introduced and have established populations in many areas, they are likely ecological generalists. Thus, species with invasion history are more likely to survive and establish in Singapore. | E.g., [4, 5, 18, 19, 39] |
| Phylogenetic |  |  |  |  |
| Family | Categorical:  25 families in total^2^ |  | Included to account for the non-independence between species due to phylogenetic relatedness. |  |

Categories for all binary attributes were either a ‘yes’ or ‘no’.

^1^www.ecopath.org

^2^See S2 File for list of fish families.

**References**

1. Yeo DCJ, Lim KKP. Freshwater ecosystems. In: Ng PKL, Corlett RT, Tan HTW, editors. Singapore biodiversity: an encyclopedia of the natural environment and sustainable development. Kuala Lumpur: Editions Didier Millet; 2011. p. 52 - 63.

2. Moyle PB, Light T. Biological invasions of fresh water: Empirical rules and assembly theory. Biological Conservation. 1996;78(1):149-61. doi: <https://doi.org/10.1016/0006-3207(96)00024-9>.

3. Huston MA, DeAngelis DL. Competition and Coexistence: The Effects of Resource Transport and Supply Rates. The American Naturalist. 1994;144(6):954-77. doi: 10.1086/285720.

4. Kolar CS, Lodge DM. Ecological predictions and risk assessment for alien fishes in North America. Science. 2002;298(5596):1233-6.

5. Moyle PB, Marchetti MP. Predicting Invasion Success: Freshwater Fishes in California as a Model. BioScience. 2006;56(6):515-24. doi: 10.1641/0006-3568(2006)56[515:PISFFI]2.0.CO;2.

6. Gin KY-H, Lin X, Zhang S. Dynamics and size structure of phytoplankton in the coastal waters of Singapore. Journal of Plankton Research. 2000;22(8):1465-84. doi: 10.1093/plankt/22.8.1465.

7. Peel MC, Finlayson BL, McMahon TA. Updated world map of the Köppen-Geiger climate classification. Hydrology and earth system sciences discussions. 2007;4(2):439-73.

8. Ruesink JL. Global analysis of factors affecting the outcome of freshwater fish introductions. Conservation Biology. 2005;19(6):1883-93.

9. Duncan RP, Bomford M, Forsyth DM, Conibear L. High predictability in introduction outcomes and the geographical range size of introduced Australian birds: a role for climate. Journal of Animal Ecology. 2001;70(4):621-32. doi: 10.1046/j.1365-2656.2001.00517.x.

10. Nico LG, Fuller PL. Spatial and Temporal Patterns of Nonindigenous Fish Introductions in the United States. Fisheries. 1999;24(1):16-27. doi: 10.1577/1548-8446(1999)024<0016:SATPON>2.0.CO;2.

11. Ehrlich PR. Attributes of invaders and the invading process: vertebrates. In: Drake JA, Mooney HA, di Castri F, Groves RH, Kruger FJ, Rejmanek M, et al., editors. Biological Invasions A Global Perspective. Chichester: John Wiley and Sons; 1989. p. 315–28.

12. Froese R, Pauly D, editors. FishBase, version (11/2014); 2014 [cited 7 April 2015]. Databse: Fishbase [Internet]. Available from: [www.fishbase.org](file:///C:\Users\dbsjoch\Dropbox\FYP\Manuscript%20draft\www.fishbase.org).

13. Deehr RA, Luczkovich JJ, Hart KJ, Clough LM, Johnson BJ, Johnson JC. Using stable isotope analysis to validate effective trophic levels from Ecopath models of areas closed and open to shrimp trawling in Core Sound, NC, USA. Ecological Modelling. 2014;282:1-17. doi: <https://doi.org/10.1016/j.ecolmodel.2014.03.005>.

14. Ng PKL, Chou LM, Lam TJ. The status and impact of introduced freshwater animals in Singapore. Biological Conservation. 1993;64(1):19-24. doi: <http://dx.doi.org/10.1016/0006-3207(93)90379-F>.

15. Ng HH, Tan HH. An annotated checklist of the non-native freshwater fish species in the reservoirs of Singapore. COSMOS. 2010;06(01):95-116. doi: doi:10.1142/S0219607710000504.

16. Duggan IC, Rixon CA, MacIsaac HJ. Popularity and propagule pressure: determinants of introduction and establishment of aquarium fish. Biological invasions. 2006;8(2):377-82.

17. Drake JM, Jerde CL. Stochastic models of propagule pressure and establishment. In: Keller RP, Lodge DM, Lewis MA, Shogren JF, editors. Bioeconomics of invasive species. New York: Oxford University Press; 2009. p. 83–102.

18. Marchetti MP, Moyle PB, Levine R. Alien fishes in California watersheds: characteristics of successful and failed invaders. Ecological Applications. 2004;14(2):587-96.

19. Marchetti MP, Moyle PB, Levine R. Invasive species profiling? Exploring the characteristics of non-native fishes across invasion stages in California. Freshwater Biology. 2004;49(5):646-61. doi: 10.1111/j.1365-2427.2004.01202.x.

20. Vila-Gispert A, Alcaraz C, García-Berthou E. Life-history traits of invasive fish in small Mediterranean streams. Biological Invasions. 2005;7(1):107-16. doi: 10.1007/s10530-004-9640-y.

21. Jeschke JM, Strayer DL. Determinants of vertebrate invasion success in Europe and North America. Global Change Biology. 2006;12(9):1608-19.

22. Knight JDM. Invasive ornamental fish: a potential threat to aquatic biodiversity in peninsular India. Journal of Threatened Taxa. 2010;2(2):700-4. doi: 10.11609/JoTT.o2179.700-4.

23. Deacon AE, Ramnarine IW, Magurran AE. How reproductive ecology contributes to the spread of a globally invasive fish. PloS one. 2011;6(9):e24416-e. Epub 2011/09/19. doi: 10.1371/journal.pone.0024416. PubMed PMID: 21957449.

24. Balon EK. Epigenesis of an epigeneticist: the development of some alternative concepts on the early ontogeny and evolution of fishes. Guelph Ichthyology Reviews. 1990;1:1-48.

25. Webb A. Risk assessment model development for establishment success and impact of non-native freshwater fishes in the Wet Tropics Bioregion, northern Queensland, Australia. A Report to the Marine and Tropical Science Research Facility (MTSRF) and Terrain Pty. Ltd. 2008 Nov. Report No. 08/23.

26. Yang SL, Tiew KN, Char CT. Artificial destratification through aeration in Upper Peirce Reservoir—Its effects on water quality and chemical costs in treatment. Public Utility Board R&D. 1993;5:32 - 49.

27. Gin KY-H, Gopalakrishnan AP. Sediment Oxygen Demand and Nutrient Fluxes for a Tropical Reservoir in Singapore. Journal of Environmental Engineering. 2010;136(1):78-85. doi: doi:10.1061/(ASCE)EE.1943-7870.0000119.

28. Feng Z. Hundreds of dead fish removed from Pandan reservoir; water quality unaffected. The Straits Times. 28 July 2014. [Cited 7 April 2015]. Available from: <https://www.straitstimes.com/singapore/environment/hundreds-of-dead-fish-removed-from-pandan-reservoir-water-quality-unaffected>.

29. Chapple DG, Simmonds SM, Wong BBM. Can behavioral and personality traits influence the success of unintentional species introductions? Trends in Ecology & Evolution. 2012;27(1):57-64. doi: <https://doi.org/10.1016/j.tree.2011.09.010>.

30. Ricciardi A, Rasmussen JB. Predicting the identity and impact of future biological invaders: a priority for aquatic resource management. Canadian Journal of Fisheries and Aquatic Sciences. 1998;55(7):1759-65. doi: 10.1139/f98-066.

31. Arthington AH, Kailola PJ, Woodland DJ, Zalucki JM. Baseline environmental data relevant to an evaluation of quarantine risk potentially associated with the importation to Australia of ornamental finfish. Report to the Australian Quarantine and Inspection Service, Department of Agriculture, Fisheries and Forestry, Canberra. 1999.

32. Stephens PA, Sutherland WJ. Consequences of the Allee effect for behaviour, ecology and conservation. Trends in Ecology & Evolution. 1999;14(10):401-5. doi: <https://doi.org/10.1016/S0169-5347(99)01684-5>.

33. Courchamp F, Berec L, Gascoigne J. Allee effects in ecology and conservation. Oxford: Oxford University Press; 2008. 272 p.

34. García‐Berthou E. The characteristics of invasive fishes: what has been learned so far? Journal of Fish Biology. 2007;71(sd):33-55.

35. Alcaraz C, Vila‐Gispert A, García‐Berthou E. Profiling invasive fish species: the importance of phylogeny and human use. Diversity and Distributions. 2005;11(4):289-98.

36. Duncan RP. Propagule pressure. In: Simberloff D, Rajmánek M, editors. Encyclopedia of Biological Invasions. Berkeley: University of California Press; 2011. p. 561–3.

37. Lim KKP, Ng PKL. A Guide to the Common Freshwater Fishes of Singapore. Singapore: Singapore Science Centre; 1990. 160 p.

38. Crooks JA. Lag times and exotic species: The ecology and management of biological invasions in slow-motion1. Écoscience. 2005;12(3):316-29. doi: 10.2980/i1195-6860-12-3-316.1.

39. Moyle PB, García-Berthou E. Fishes. In: Simberloff D, Rajmánek M, editors. Encyclopedia of Biological Invasions. Berkeley: University of California Press; 2011. p. 229–34.
