# Supplementary material for: Invasive species trait-based risk assessment for non-native freshwater fishes in a tropical city basin in Southeast Asia
Source: PLoS One. 2021 Mar 16;16(3):e0248480. doi: 10.1371/journal.pone.0248480 (PMC7963036; doi:10.1371/journal.pone.0248480)
Supplement: S2 File — (DOCX) [file pone.0248480.s003.docx]

**S2 File. List of families of non-native freshwater fishes in Singapore, summary of data gathered for the 20 species attributes used in the trait-based risk assessment, variable importance values and predictions from Random Forest analyses**

**Table A. List of fish families (total= 25) of introduced freshwater fishes (n = 98) in Singapore [1].**

| **Family*** | **Number of species** |
| --- | --- |
| Cyprinidae | 25 |
| Cichlidae | 23 |
| Osphronemidae | 8 |
| Poeciliidae | 8 |
| Characidae | 4 |
| Bagridae | 3 |
| Loricariidae | 3 |
| Osteoglossidae | 3 |
| Gobiidae | 2 |
| Lepisosteidae | 2 |
| Mastacembelidae | 2 |
| Notopteridae | 2 |
| Ambassidae | 1 |
| Aplocheilidae | 1 |
| Arapaimatidae | 1 |
| Botiidae | 1 |
| Callichthyidae | 1 |
| Channidae | 1 |
| Clariidae | 1 |
| Datnioididae | 1 |
| Eleotridae | 1 |
| Mochokidae | 1 |
| Pangasiidae | 1 |
| Pimelodidae | 1 |
| Potamotrygonidae | 1 |

**Table B**. **Summary of information gathered for 20 species attributes (ecological, biological and behavioural, human uses, and historical).** Mean values and standard errors (in parenthesis) are given for continuous data, and total count value for categorical data.

| **Attributes** | **Categories** | | **Distribution of values** | | | |
| --- | --- | --- | --- | --- | --- | --- |
|  |  |  | **Established**  **(n= 36)** | | **Failed to establish**  **(n=24)** | |
| **Ecological** |  | |  | |  | |
| Habitat Type | Lentic | | 3 | | 1 | |
|  | Lotic slow | | 4 | | 6 | |
|  | Lotic fast | | 2 | | 4 | |
|  | Both (Lentic and lotic) | | 27 | | 13 | |
|  |  | |  | |  | |
| Habitat Generalist | Yes | | 33 | | 19 | |
|  | No | | 3 | | 5 | |
|  |  | |  | |  | |
| Vertical Position | Benthopelagic | | 30 | | 21 | |
|  | Pelagic | | 1 | | 2 | |
|  | Demersal | | 5 | | 1 | |
| Habitat Salinity | Freshwater | 14 | | 17 | |  |
|  | Both (freshwater and brackish) | 22 | | 7 | |  |
| Climate Match | Yes | 26 | | 9 | |  |
|  | No | 10 | | 15 | |  |
|  |  |  | |  | |  |
| Climate Types |  | 3.3 (0.18) | | 3.4 (0.33) | |  |
|  |  |  | |  | |  |
| Trophic Level |  | 3.11 (0.105) | | 2.73 (0.111) | |  |
|  |  |  | |  | |  |
| **Biological/ Behavioural** |  |  | |  | |  |
| Maximum Standard Length (mm) |  | 387.7 (62.4) | | 434.7 (93.6) | |  |
|  |  |  | |  | |  |
| Absolute Fecundity |  | 27,654 (12,363) | | 339,633 (129,476) | |  |
|  |  |  | |  | |  |
| Mode of Reproduction | Oviparous | 32 | | 19 | |  |
|  | Ovoviviparous | 4 | | 5 | |  |
|  |  |  | |  | |  |
| Parental Care | Non-guarders | 11 | | 10 | |  |
|  | Guarders | 16 | | 8 | |  |
|  | Bearers | 9 | | 6 | |  |

**Table B.** **(continued) Summary of information gathered for 20 species attributes (ecological, biological and behavioural, human uses, and historical).** Mean values and standard errors (in parenthesis) are given for continuous data, and total count value for categorical data.

| **Attributes** | **Categories** | **Distribution of values** | | |
| --- | --- | --- | --- | --- |
|  |  | **Established**  **(n= 36)** | **Failed to establish**  **(n=24)** | |
| **Biological/ Behavioural** |  |  | |  |

| Diet | Herbivorous | 1 | 2 |
| --- | --- | --- | --- |
|  | Omnivorous | 26 | 20 |
|  | Carnivorous | 9 | 2 |
|  |  |  |  |
| Air-Breathing | Yes | 8 | 7 |
|  | No | 28 | 17 |
|  |  |  |  |
| Adult Gregarious | Yes | 22 | 18 |
|  | No | 14 | 6 |
| **Human use** |  |  |  |
| Aquarium | Yes | 33 | 17 |
|  | No | 3 | 7 |
|  |  |  |  |
| Aquaculture | Yes | 10 | 10 |
|  | No | 26 | 14 |
|  |  |  |  |
| Angling | Yes | 3 | 0 |
|  | No | 33 | 24 |
|  |  |  |  |
| Biological Control | Yes | 3 | 1 |
|  | No | 33 | 23 |
| **Historical** |  |  |  |
| Year of Introduction |  | 1972 (5.56) | 1968 (4.98) |
|  |  |  |  |
| Invasion History | Yes | 27 | 12 |
|  | No | 9 | 12 |


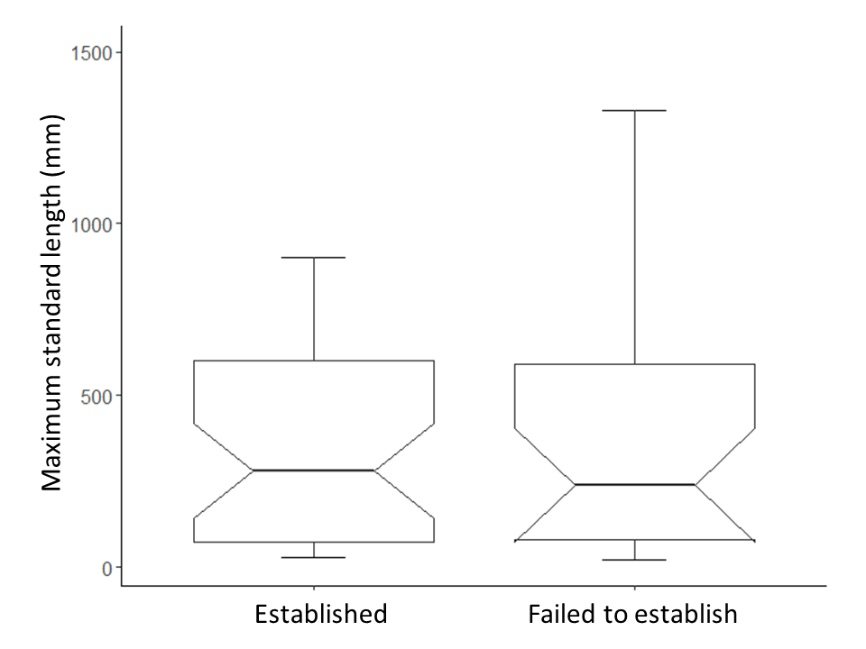


**Figure A**. Notched boxplots of maximum standard length (mm) of non-native freshwater fishes that established (n=36) and failed to establish (n=24) in Singapore.


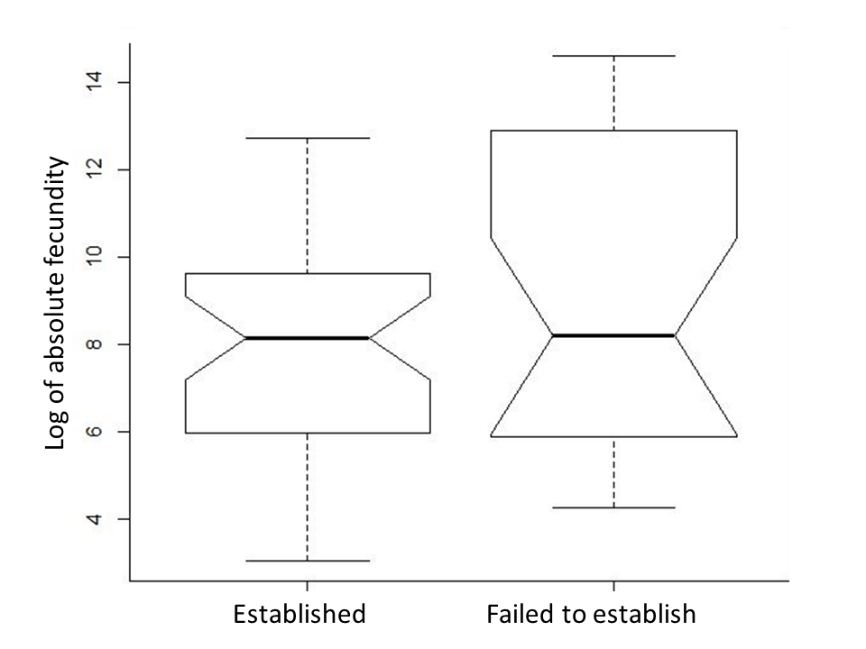


**Figure B**. Notched boxplots of log-transformed absolute fecundity of non-native freshwater fishes that established (n=36) and failed to establish (n=24) in Singapore.

**Figure C**. Proportion of non-native freshwater fishes that provide or do not provide parental care.

**Table C**. Variable importance of each species attribute obtained through random forest analyses. The full model contained all 21 attributes while the reduced model excluded the attributes year of introduction and invasion history. Models constructed using 58 species were used to check for model reliability by comparing the model predictions with new establishment records.

|  | Variable importance value | | | | | |
| --- | --- | --- | --- | --- | --- | --- |
| Attribute | **Full model** | | | **Reduced model** | | |
| Number of species analysed | **60 species** | | **58 species** | **60 species** | | **58 species** |
| Climate match | 1.64E-02 | 1.30E-02 | | 2.05E-02 | 9.05E-03 | |
| Invasion history | 8.23E-03 | -1.19E-03 | | - | - | |
| Absolute fecundity | 8.18E-03 | 8.43E-03 | | 8.77E-03 | 1.11E-02 | |
| Trophic level | 2.59E-03 | 2.95E-03 | | 1.36E-03 | 3.90E-03 | |
| Aquarium | 1.36E-03 | -1.90E-04 | | 9.09E-04 | 1.71E-03 | |
| Air-breathing | 1.09E-03 | 1.90E-04 | | -7.27E-04 | -5.71E-04 | |
| Family | 1.05E-03 | 8.57E-04 | | 1.23E-03 | 3.33E-03 | |
| Habitat salinity | 9.55E-04 | -1.48E-03 | | -6.82E-04 | -1.43E-03 | |
| Climate types | 7.73E-04 | -7.14E-04 | | -1.86E-03 | -9.52E-04 | |
| Parental care | 7.27E-04 | -1.05E-03 | | 9.09E-05 | 9.52E-05 | |
| Year of introduction | 5.91E-04 | -1.57E-03 | | - | - | |
| Mode of reproduction | 5.45E-04 | -5.55E-20 | | -1.82E-04 | 5.71E-04 | |
| Adult gregarious | 3.64E-04 | 5.24E-04 | | -5.45E-04 | 1.43E-03 | |
| Diet | 2.73E-04 | 0.00E+00 | | 9.09E-05 | 9.52E-05 | |
| Habitat type | 2.27E-04 | 1.05E-03 | | 2.73E-04 | 1.33E-03 | |
| Aquaculture | 9.09E-05 | -6.67E-04 | | -6.36E-04 | 1.71E-03 | |
| Maximum standard length | 5.55E-20 | 0.00E+00 | | 1.14E-03 | -2.86E-04 | |
| Water column | 5.55E-20 | 0.00E+00 | | 0.00E+00 | -9.52E-05 | |
| Angling | 0.00E+00 | 0.00E+00 | | 0.00E+00 | 0.00E+00 | |
| Biological control | 0.00E+00 | 0.00E+00 | | 0.00E+00 | 0.00E+00 | |
| Habitat generalist | 0.00E+00 | 0.00E+00 | | 1.82E-04 | 0.00E+00 | |

**Table D**. Probability of successful establishment of five species recorded to have established since 2016 [1, 2], predicted using the full and reduced (exclusion of attributes year of introduction and invasion history) random forest models. Both models had high accuracy with all species correctly predicted to be able to establish.

|  |  | **Probability of establishing** | |
| --- | --- | --- | --- |
| **Species** | **Common name** | **Full model** | **Reduced model** |
| *Apistogramma borellii* | Umbrella dwarf cichlid | 0.675 | 0.713 |
| *Betta splendens* | Siamese fighting-fish | 0.602 | 0.645 |
| *Cichla temensis* | Speckled pavon | 0.807 | 0.851 |
| *Hemigrammus rodwayi* | Gold tetra | 0.657 | 0.679 |
| *Pterygoplichthys joselimaianus* | Spotted sailfin catfish | 0.649 | 0.666 |

**Table E.** Comparison of the five important attributes between cichlids and cyprinids that successfully established (ES) and failed to establish in Singapore (Failed ES).

| **Family** | **Status** | **n** | **Invasion history (yes)** | **Climate match (yes)** | **Absolute fecundity** | **Trophic level** | **Use in aquarium (yes)** |
| --- | --- | --- | --- | --- | --- | --- | --- |
| Cichlidae | ES | 11 | 72.7% | 72.7% | 4,103.00 | 3.03 | 90.9% |
|  | Failed ES | 5 | 60.0% | 60.0% | 4,467.00 | 2.54 | 60.0% |
| Cyprinidae | ES | 10 | 70.0% | 70.0% | 39,896.00 | 3.1 | 80.0% |
|  | Failed ES | 8 | 62.5% | 12.5% | 890,098.00 | 2.39 | 37.5% |

References

1. Ng HH, Tan HH. An annotated checklist of the non-native freshwater fish species in the reservoirs of Singapore. COSMOS. 2010;06(01):95-116. doi: doi:10.1142/S0219607710000504.

2. Tan HH, Lim KKP, Liew JH, Low BW, Lim RBH, Kwik JTB, et al. The non-native freshwater fishes of Singapore: an annotated compilation. Raffles Bulletin of Zoology. 2020;68:150-95. doi: 10.26107/RBZ-2020-0016.
